# Awareness and knowledge of physicians and residents on the non-sexual routes of human papilloma virus (HPV) infection and their perspectives on anti-HPV vaccination in Jordan

**DOI:** 10.1371/journal.pone.0291643

**Published:** 2023-10-11

**Authors:** Arwa Qaqish, Nour Abdo, Manal Mohammad Abbas, Nour Saadeh, Mohammad Alkhateeb, Renad Msameh, Shahd Tarawneh, Mona Al-Masri

**Affiliations:** 1 Department of Biology and Biotechnology, Faculty of Science, The Hashemite University, Zarqa, Jordan; 2 Department of Cellular Therapy and Applied Genomics, King Hussein Cancer Center (KHCC), Amman, Jordan; 3 Department of Public Health, Community Medicine and Family Medicine, Jordan University of Science and Technology, Irbid, Jordan; 4 Department of Medical Laboratory Sciences, Faculty of Allied Medical Sciences, Al-Ahliyya Amman University, Amman, Jordan; 5 The National Center for Diabetes, Endocrinology and Genetics, Amman, Jordan; 6 Faculty of medicine, The Hashemite University, Zarqa, Jordan; 7 Department of General Surgery, King Abdullah University Hospital (KAUH), Jordan University of Science and Technology, Irbid, Jordan; Jordan University of Science and Technology, JORDAN

## Abstract

**Background and objectives:**

Although penetrative sex is the most common route of HPV infection, there is strong evidence of non-sexual modes of transmission. As the first of its kind, this study aimed to investigate the knowledge and awareness of Jordanian physicians on such routes.

**Methods:**

A questionnaire was conducted among a national Jordanian sample of physicians from Jordanian health sectors. The survey included questions assessing participants’ knowledge on HPV, non-sexual routes of infection and HPV vaccines. Physicians’ attitudes towards HPV screening and vaccination were covered. Statistical analysis was carried out using SAS 9.4, ANOVA, post-hoc Tukey-Honest test and Kruskal-Wallis test. All significant differences were set at α = 0.05.

**Results:**

A total of 412 participants completed the survey. Physicians showed a huge deficit in knowledge on nonsexual routes of HPV transmission. They agreed that the most and least common routes of non-sexual transmission are skin to mucosa (64%) and contaminated water (15%), respectively. Females showed significantly better knowledge in all aspects of HPV transmission and vaccination (p<0.0001) and more positive attitudes towards HPV screening and vaccination compared to males (p = 0.03). Age group ≤ 25 and academic physicians demonstrated higher knowledge on HPV vaccines compared to their counterparts in non-academic places (p = 0.002). Specialty and experience seemed to have no impact on knowledge or attitudes of participants. Higher knowledge physicians had more positive attitude towards vaccination and screening compared to lower knowledge fellows (p<0.001).

**Conclusions:**

The noteworthy findings of this study is the extremely low level of knowledge on non-sexual routes of HPV infection among Jordanian physicians. Increasing the level of awareness of physicians and healthcare workers on these routes and their association with cervical and other cancers through university curricula and other reliable sources is strongly recommended.

## 1. Introduction

Human papillomavirus (HPV) is a non-enveloped epitheliotropic double stranded DNA virus from the *Papillomaviridae* family. There are over 200 genotypes of HPV. Fourty of these belong to the α genus, 15 of which are considered high-risk (HR)-HPV types as they can lead to carcinogenesis [1].

HPV is estimated to cause 570,000 cases of cancer in women and 60,000 cases of cancer in men worldwide every year. Globally, HPV is the cause of 99% of cervical cancers, 90% of anal cancers, 70% of vulvar and vaginal cancers, 60% of penile cancers and 70% of oropharyngeal cancers, making up more than half of all malignancies related to infections [2–5].

HPV infection can be asymptomatic and clear on its own or can cause a wide spectrum of symptoms ranging from a group of benign lesions such as skin and genital warts to the formation of malignancies [6]. Beside the genetic type, it has been established that a few factors including the host genetic background, smoking, alcohol consumption, the presence of co-infections and the age of sexual debut affect the clearance time of the virus [7].

Penetrative and non-penetrative sex are the main routes of HPV transmission through skin-to-skin or skin-to-mucosa contact [8]. HPV can be transmitted from genitals to hands or hands to genitals through both sexes [9]. Many non-sexual routes have been reported to cause HPV transmission as evidenced by the detection of the virus in female virgins and genital warts in children who have never experienced sexual abuse [10, 11]. Horizontal transmission of HPV involves contact with the mouth, fingers, and skin (other than sexual). Studies suggest that self-inoculation is a possible HPV transmission route [8, 9]. Another HPV transfer route is vertical transmission from mother to fetus through the amniotic fluid and/or placenta and through contact with mother’s mucosa during natural birth [12, 13]. Mother to newborn transmission can occur through skin-to-skin contact. Water is another potential source of HPV infection. Although waterborne HPV transmission has never been proven, HPV DNA has been found in sewage water, rivers and swimming pools [14–16]. Importantly, HPV was detected on medical instruments, such as ultrasound probes, making these tools powerful candidates of transmission [17].

Three HPV vaccines were licensed since 2006. The bivalent Cervarix targeting HR HPV16 and HPV18. The quadrivalent Gardasil protecting against LR HPV6 and HPV11 and HR HPV16 and HPV18. The nanovalent Gardasil 9 targets 5 other HR types, namely HPV31, HPV33, HPV45, HPV52, and HPV58. All three vaccines proved to significantly reduce the development of premalignant and malignant lesions and decrease anogenital infections in women [18].

HPV infection rates vary in different countries, with a global prevalence of 11.7% and highest infection rates recorded in Africa (21%) [19]. In Arab countries, HPV prevalence rates varied from low to moderate as reported in Kuwait (2.4%, 2007) [20], Qatar (9.8%, 2014) [21], Saudi Arabia (9.8%, 2011) [22], Bahrain (9.8%, 2011) [23], and Palestine (13%, 2007) [24].

In Jordan, data and updates on the prevalence of HPV infection are deficient [25]. In 2017, a study done at King Hussein Cancer Center showed a 3.8% abnormal Papanicolaou (Pap) smear rate among a total of 5,529 routine tested smears. This study argued against the implementation of population HPV testing and vaccination based on the low prevalence of cervical cancers in the country [26]. In 2020, the prevalence of HPV infection in Jordan was reported as 4% among women attending the Gynecology Clinic at Prince Hamza Hospital (Amman, Jordan) with HPV-16 being the most common subtype (43%) [27].

During 2000–2013, cervical cancer was ranked the 10th most common cancer among women aged 15–44 years in the country [28]. In 2012, the annual incidence of cervical cancer was estimated at 50–60 cases with a 50% mortality rate [29]. Interestingly, this number doubled 9 years later. In 2021, the HPV Information Centre (October 2021) reported an annual cervical cancer diagnosis of 115 women with 71 deaths [30]. This might indicate an increased rate of HPV transmission in the country. Recent studies have shown that ~ 90% of screened cervical cancers were HPV DNA +ve, with HPV-16 followed by HPV-18 as most prevalent [31, 32]. As for head and neck cancers, the prevalence of HPV was reported at 14.8% and 31.1% in two recent studies in Jordan, with HPV-16 being the significantly predominant strain [33, 34].

Reports on the rate of cervical smear test uptake in Jordan are scarce. Despite a slow improvement in recognizing the importance of the test and the rate of screening among Jordanian women including healthcare workers over time, all reported low rates of test uptake, with the highest reaching 38% of female study participants. These reports identified knowledge deficiency on the existence of the test, not being referred to undergo the test by a health professional and fear of unfavorable results as major obstacles faced by Pap smearing in the country. All reached to same conclusion of a pressing need to implement a national screening program advertised by health education through the media. [35, 36].

Several studies assessed knowledge on HPV infection, HPV caused cancers, and attitudes towards HPV vaccination in Jordan. Target participants included students (health sciences, medicine and dentistry), women visiting clinics, dentists and gynecologists. Although awareness of HPV infection, risks of HPV related cancers (especially other than cervical), Pap screening, presence of HPV vaccines and their protective potential varied among investigations, with older medical students and physicians being more knowledgeable, all studies reported a deficit in knowledge and a relative resistance to vaccination [37]. The main factors for HPV vaccine rejection included cost, decreased risk to get HPV infection and complacency [37–42]. These findings urged the implementation of intervention measures including curricular changes, training workshops and awareness campaigns.

Undoubtedly, there is a religious and cultural based social discomfort in Jordan and Muslim countries towards HPV and sexually transmitted diseases. This led to the hindering of HPV vaccination and screening. We believe that an understanding of potential non-sexual HPV transmission could relief such discomfort and could help increase rates of vaccine acceptance. We hypothesize that Jordanian physicians have low knowledge on non-sexual routes of HPV transmission and therefore do not encourage anti-HPV vaccination and/or HPV screening. Hence, this study aimed at investigating the knowledge and awareness of Jordanian physicians on the non-sexual routes of HPV transmission and to correlate such knowledge to their attitude towards HPV vaccination and screening.

## 2. Methods

### 2.1. Study design

A self-administered electronic questionnaire was conducted among a national Jordanian sample of physicians, residents and general practitioners (GP). The questionnaire was distributed to all health sectors, including Ministry of Health (MOH), the Royal Medical Services (RMS), university hospitals (Academia), and private practice.

The survey was developed and presented to participants in English. Before conducting the pilot test, the face validity of each item was checked by the authors. The instrument was validated in a pilot test with ten physician respondents who were not included in the final analysis. A minor revision of some items was done following the pilot test.

We used the following formula to obtain the minimal sample size needed for the study:

$$n={\left(z_{\alpha /2}\right)}^{2}pq/D^{2}$$

Where z is the normal standard deviation which equals 1.96 for a 95% confidence level, p is the expected prevalence of the condition of interest, q equals 1-p, and D is the margin of error acceptable to the researcher. Assuming a prevalence of 50% and a margin of error of 5%, and a 95% CL, the needed sample size was 384 participants. The study had more than 400 participants.

Accurate targeting of the respondents was done through the authors’ direct contact with physicians and residents in medical hospitals. Also, it was distributed among the author’s direct contacts and on different social media platforms (Facebook and WhatsApp). The survey distribution took place in August 2022, with no incentives for participation. Informed consent was added in the introductory section of the survey to assure the respondents of their anonymity, agreement to participate in the study upon answering the questions, and their right to withdraw from the study at any time.

### 2.2. Ethical approval

The study was approved by the Department of Biology and Biotechnology/Faculty of science/ Hashemite University (No.22/7/2021/2022).

### 2.3. Survey items

The survey includes four sections: the first section represents an invitation to participate in the survey by displaying its aim, assuring the participant’s agreement in participation, along with their right of withdrawal and anonymity. The second section comprises nine items assessing various characteristics of the participants (gender, marital status, occupation, specialty, place of work, previous training or published research on sexually transmitted diseases, and the frequency of encountered patients diagnosed with HPV).

The third section comprises an introduction regarding participants’ general knowledge of HPV. The section starts with yes or no questions concerning whether they have heard of HPV before the survey, asking if HPV was common in Jordan, and whether HPV infection could be asymptomatic. Their knowledge of whom HPV infects was covered (males, females, or both). Asking about HPV routes of transmission was included with check boxes of yes and no near each possible route (sexual intercourse, skin-skin contact, skin-mucosa contact, mother to fetus, contact with contaminated medical equipment, contact with contaminated water (swimming pools), self-inoculation, and none). The most adverse possible health outcomes of HPV infection for males and females were added for the participants to choose from. Last but not least, this section covered HPV treatment availability, recommendations for regular testing for females and high school students in Jordan, and the issue of lack of knowledge regarding HPV among the community and healthcare workers.

The final section of the survey covered HPV vaccination. The section incorporated yes or no questions regarding the presence of a vaccine, whether it is protective in case of a current HPV infection, its availability in Jordan, and whether the participants agree that the vaccination should be obligatory in Jordan. The percentage of cervical cancer prevention by vaccination was covered and followed by the best time to receive the vaccine (during childhood or puberty, prior to starting sexual activity, or at any age). Also, the participants were asked if they would take the vaccine if available and whether they would recommend it to their patients, with reasons to choose from for those who disagree. See S1 Table.

### 2.4. Knowledge score calculation

A team of authors evaluated the relevance and representativeness of the current questionnaire’s content validity. To assess the overall knowledge of the participants regarding HPV and its vaccine, we used three HPV knowledge (K) scores (HPV K-General (G), HPV K-NonSexual (NS), HPV K-Vaccine (V)). HPV K-G includes the following five points: 1) ability of HPV to infect both females and males; 2) asymptomatic HPV infections are possible; 3) HPV transmission through sexual routes 4) cancer is a serious consequence of HPV infection in both men and women; 5) HPV infections can be treatable. Regarding the HPV K-NS, it also consists of five points: 1. transmission of HPV through skin to skin or skin to mucosa contact; 2. mothers being a source of HPV in neonates; 3. contaminated medical equipment’s capacity to spread HPV to patients; 4. HPV spread is accelerated by self-inoculation; 5. presence of HPV in water. Finally, the HPV K-V score encompasses four points: 1- existence of HPV vaccine; 2- protectiveness of the vaccine against HPV associated cancers in those already infected with certain strains of HPV; 3- percentage of protection from malignancies attributable to HPV that the vaccine confers; 4- age group that benefits most from vaccination. The maximum possible score was 6, 5, and 4 according to the HPV K-G, K-NS, K-V respectively while the minimum was zero for all three of them. See S2 Table for detailed scoring.

### 2.5. Attitude score calculation

The physicians’ attitude towards regular screening for HPV in the community as well as their perspective and recommendation of HPV vaccine was assessed using the attitude scale (A-Scale). Four points were evaluated: 1) The willingness of the participants to receive the HPV vaccine for free; 2) The confidence to suggest the vaccine to their patients; 3) The necessity of routine HPV screening among young adults in the community; 4) The significance of regular HPV screening among high schoolers. Each of the above was assessed with a dichotomous scale (“Yes” or “No”) and given 1 point. Thus, the maximum score was 4 and the minimum was zero. Higher Attitude scale values indicated a more positive attitude of the physician toward advising regular testing of HPV and promoting HPV vaccine. See S2 Table for detailed scoring.

### 2.6. Statistical analysis

Statistical analysis was carried out using SAS 9.4 (Cary, NC, USA). Descriptive statistics were used to describe demographic and general characteristics of participants. Percentages and frequencies were used to describe categorical variables, while mean and range or median and inter-quartile range (IQR) were calculated to describe continuous variables. ANOVA was used to compare the differences between mean scores of age groups, gender (males vs. females), specialty (surgery, internal medicine, pediatrics, gynecology, or others), experience (specialist, resident, general practitioner), and workplace (Ministry of Health, academia, private sector, military services) for each one of the following variables about HPV, knowledge about non-sexual routes, knowledge about HPV-vaccine, total knowledge scores, and attitude scores. Post-hoc Tukey-Honest test was run for significant associations to identify which pairs where significant after adjustment for p-values. Because of the data nature of scores, we also ran Kruskal-Wallis test to compare differences between median scores of age groups, gender, specialty, experience, and workplace for each one of the following variables about HPV, knowledge about non-sexual routes, knowledge about HPV-vaccine, total knowledge scores, and attitude scores. All significant differences were set at α = 0.05.

## 3. Results

### 3.1. Sociodemographic characteristics of respondents

A total of 412 participants completed the survey; 158 participants from the MOH (38.4%), 128 from academia (31.1%), 105 were from private practices (25.5%), and 21 from the RMS (5.1%). More than half of study participants were females (53.2%) and unmarried (54.8%). The age group 26–40 was dominant (71.8%) (Table 1).

**Table 1:** Characteristic of participants in “Awareness and knowledge of physicians and residents in the non-sexual routes of HPV infection and their perspective on anti-HPV vaccination in Jordan” study, 2022.

Characteristic	Number	%
**Gender**		
Female	219	53.2
Male	193	46.8
**Marital Status**		
Unmarried	226	54.8
Currently married	186	45.2
**Age**		
≤ 25	75	18.2
26–40	296	71.8
41–60	31	7.5
above 60	10	2.4
**Occupation**		
Specialist	130	31.6
Resident	215	52.2
General practitioner	67	16.3
**Specialty**		
Surgery	60	14.6
Internal Medicine	46	11.2
Pediatrics	25	6.1
Gynecology	72	17.5
Others *	209	50.7
**Work**		
Ministry of health	158	38.4
Academia	128	31.1
Private sector	105	25.5
Military or Royal Medical Services	21	5.1

*Others: Family Medicine 36, Dermatology 17, Pathology 11, Urology 9, Anesthesiology 8, Ophthalmology 7, Orthopedics 5, Otorhinolaryngology 2, Public health 2, Anatomy 1, Forensic Medicine 1, Neurology 1, Pharmacology 1, Radiology 1, General practitioners 67, missing data 40

In terms of specialty, gynecologists, surgeons, internists and pediatricians comprised 17.5%, 14.6%, 11.2% and 6.1% of the participants, respectively. The remaining specialties (named others) comprised 50.7% (detailed in Table 1). In terms of experience level, resident physicians (52%) were more prevalent compared to specialists (31.6%) and general practitioners (16.3%).

### 3.2. Overall level of HPV knowledge among participants who have heard of the virus

Overall, 97.8% of all participants stated they heard of HPV before encountering the questionnaire. Male participants were less likely to have general knowledge about HPV compared to their female colleagues (p = 0.005). No significant differences were found in K-HPV general scores of physicians based on age, specialty and experience (respectively; p = 0.117, p = 0.455, p = 0.781).

As female participants showed more general knowledge than males, they also evinced more knowledge on non-sexual routes of HPV transmission and higher knowledge on anti-HPV vaccination (p = 0.022, p = 0.000).

Age group ≤ 25, workers in academia and RMS demonstrated higher knowledge scores on anti-HPV vaccines than other age and workplace groups (p = 0.013, p = 0.000).

The HPV total-score was used to evaluate the overall general, non-sexual routes and vaccines knowledge of physicians per age, gender, specialty, experience and workplace. The total HPV was higher among female physicians compared to their male counterparts (p<0.0001) and also in academic physicians in comparison to their colleagues in other workplaces (p<0.001). In addition, youngest physicians (≤25 years old) scored higher than older age groups (p = 0.04). Notably, specialty and years of experience demonstrated no impact on HPV knowledge in total (Tables 2 and 3).

**Table 2:** Human papillomavirus (HPV) knowledge scores stratified by HPV general knowledge (K-HPV general), knowledge on non-sexual routes (K-nonsexual routes) of HPV transmission and knowledge on anti-HPV vaccines (K-HPV vaccine) based on participants age, gender, specialty, experience, and workplace.

Characteristic	K- HPV General			K- Nonsexual Routes			K- HPV Vaccine		
Age	Mean (Range)	Median (IQR)	p-Value *	Mean (Range)	Median (IQR)	p-Value *	Mean (Range)	Median (IQR)	p-value *
≤ 25 (75)	4.7 (2–6)	5.0 (4–5)		2.2 (0–5)	2.0 (1–3)		2.5 (0–4)	3.0 (2–3)	
26–40 (296)	4.5 (1–6)	5.0 (4–5)	0.117	2.2 (0–5)	2.0 (1–3)	0.509	2.0 (0–4)	2.0 (1–3)	0.013
41–60 (31)	4.6 (2–6)	5.0 (4–5)	0.109	2.5 (0–5)	2.5 (1.5–3.5)	0.547	2.0 (0–4)	2.5 (1–3)	0.010
above 60 (10)	4.0 (2–5)	4.0 (4–5)		1.8 (0–3)	2.0 (1–3)		1.8 (0–3)	2.0 (2–2)	
**Gender**									
Female (219)	4.7 (2–6)	5.0 (4–5)	0.005	2.4 (0–5)	2.0 (1–3.5)	0.022	2.3 (0–4)	2.0 (2–3)	0.000
Male (193)	4.4 (1–6)	4.0 (4–5)	0.005	2.0 (0–5)	2.0 (1–3)	0.021	1.9 (0–4)	2.0 (1–3)	0.000
**Specialty**									
Surgery (60)	4.8 (3–6)	5.0 (4–5)		2.0 (0–5)	2.0 (1–3)		1.7 (0–4)	2.0 (0–3)	
Internal Medicine (46)	4.5 (2–6)	5.0 (4–5)	0.455	2.0 (0–5)	2.0 (1–3)	0.128	2.2 (0–4)	2.0 (2–3)	0.059
Pediatrics (25)	4.4 (2–6)	4.5 (3.5–5)	0.723	1.8 (0–5)	1.8 (0–3)	0.108	2.1 (0–4)	2.0 (2–3)	0.124
Gynecology (72)	4.6 (3–6)	5.0 (5–4)		2.4 (0–5)	2.5 (1–3.3)		2.3 (0–4)	3.0 (2–3)	
Others (102)	4.6 (2–6)	5.0 (4–5)		2.3 (0–5)	2.0 (1–3.5)		2.1 (0–4)	2.0 (2–3)	
**Experience**									
Specialist (130)	4.6 (2–6)	5.0 (4–5)		2.3 (0–5)	2.0 (1–3)		2.2 (0–4)	2.0 (2–3)	
Resident (215)	4.5 (1–6)	5.0 (4–5)	0.781	2.1 (0–5)	2.0 (1–3)	0.397	2.0 (0–4)	2.0 (1–3)	0.442
General practitioner (67)	4.5 (2–6)	5.0 (4–5)	0.760	2.2 (0–5)	2.0 (1–3.5)	0.390	2.0 (0–4)	2.0 (2–3)	0.386
**Work**									
Ministry of health (158)	4.4 (2–6)	4.0 (4–5)	0.045	2.0 (0–5)	2.0 (1–3)	0.127	1.7 (0–4)	2.0 (0–3)	0.000
Academia (128)	4.7 (2–6)	5.0 (4–5)	0.031	2.4 (0–5)	2.0 (1–3.5)	0.135	2.4 (0–4)	3.0 (2–3)	0.000
Private sector (105)	4.6 (1–6)	5.0 (4–5)		2.3 (0–5)	2.0 (1–3)		2.1 (0–4)	2.0 (2–3)	
Military Services (21)	4.3 (3–5)	4.0 (4–5)		2.4 (0–5)	2.0 (1–3.5)		2.4 (0–4)	3.0 (2–3)	

*The first p-value shows the ANOVA results for differences between means, while the second p-value shows the difference between medians using Kruskal-Wallis test.

**Table 3:** Human papillomavirus (HPV) total knowledge (K-total) scores based on participants’ age, gender, specialty, experience, and workplace.

Characteristic	K-Total Score		
Age	Mean (Range)	Median (IQR)	p-Value*
≤ 25 (75)	9.4 (4–14)	9.5 (8–11)	
26–40 (296)	8.7 (1–14.5)	9 (7–10.5)	0.0419
41–60 (31)	9.2 (4–13)	9.5 (8–11)	0.0502
above 60 (10)	7.6 (5–10)	8.5 (5–9)	
**Gender**			
Female (219)	9.3 (4–14)	9 (8–11)	< .0001
Male (193)	8.3 (1–14.5)	8 (6–10)	< .0001
**Specialty**			
Surgery (60)	8.5 (4–13)	8.5 (7–10.3)	
Internal Medicine (46)	8.7 (3–14)	9 (7–10.5)	0.194
Pediatrics (25)	8.3 (4–14)	8 (6–10.3)	0.198
Gynecology (72)	9.3 (5–14)	9.0 (8–11)	
Others (102)	9.0 (4–14)	9.0 (7–11)	
**Experience**			
Specialist (130)	9.1 (3–14)	9.5 (8–10.5)	0.2648
Resident (215)	8.7 (1–14.5)	9 (7–10.5)	0.1655
General practitioner (67)	8.7 (3–14)	9 (7–10.5)	
**Work**			
Ministry of health (158)	8.2 (4–14)	8 (6.5–9.5)	
Academia (128)	9.5 (4–14.5)	10 (8–11)	< .0001
Private sector (105)	9 (1–14)	9 (7–11)	< .0001
Military Services (21)	8.9 (4.5–12.5)	9 (8–10.5)	

*The first p-value shows the ANOVA results for differences between means, while the second p-value shows the difference between medians using Kruskal-Wallis test.

### 3.3. Physicians’ knowledge on different non-sexual routes of HPV infection was extremely low

**Fig 1:**
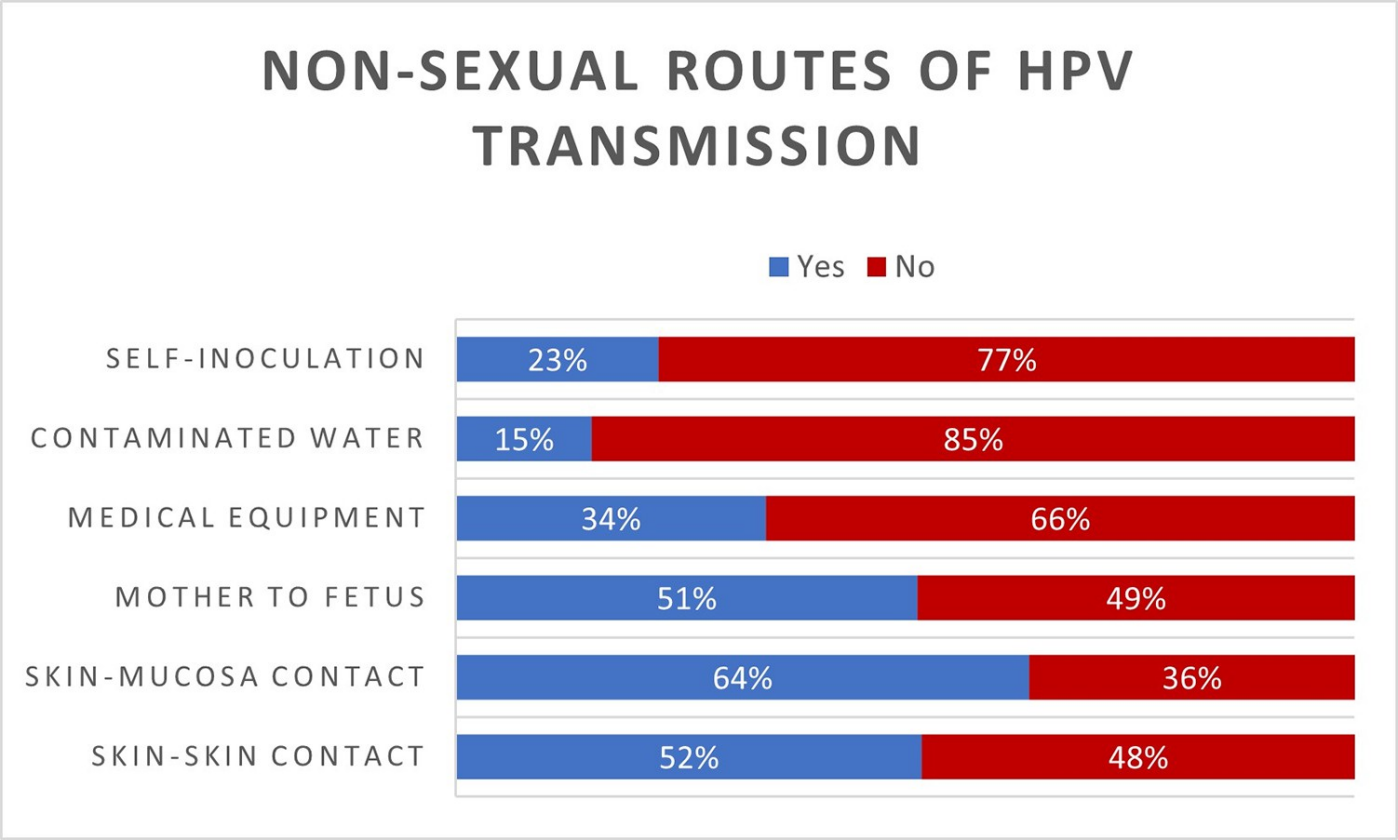
The prior knowledge of the nonsexual routes of HPV transmission among the study participants.

Although skin to mucosa and skin to skin contact are means of both sexual and nonsexual transmission of HPV, only 64% and 52% of participants respectively, agreed that these could form an HPV transmission route. Physicians’ belief in contaminated water (15%), self-inoculation 23%), medical equipment (34%) and mother to fetus (51%) as potential routes of HPV transmission is extremely low (Fig 1).

### 3.4. Overall attitude of participants who have heard of the virus towards HPV vaccination

As shown in Table 4, youngest physicians (≤ 25 years old) scored the highest in terms of attitude towards HPV vaccination and screening compared to other age groups (p = 0.002). The trend shown here demonstrates more positive attitude in young physicians (≤40 years old) compared to older ones (≥41 years old). Females showed significantly more positive attitude than males (p = 0.03). Practice type (specialty) had no effect on the physicians’ attitude towards vaccination and screening, but years of experience proved to significantly impact attitude scores with GPs being more positive that residents and specialists. Attitude of academic physicians did not differ from that of their counterparts in MOH, private sector and MRS.

**Table 4:** Human papillomavirus (HPV) attitude scores based on participants age, gender, specialty, experience, and workplace.

Characteristic	Attitude Score		
Age	Mean (Range)	Median (IQR)	P-Value
≤ 25 (75)	2.8 (0–4)	3.0 (2–3)	
26–40 (296)	2.6 (0–4)	3.0 (2–3)	0.002
41–60 (31)	1.9 (0–4)	2.0 (2–3)	0.009
above 60 (10)	2.2 (0–4)	2.0 (2–3)	
**Gender**			
Female (219)	2.7 (0–4)	3.0 (2–4)	0.030
Male (193)	2.4 (0–4)	2.0 (2–3)	0.023
**Specialty**			
Surgery (60)	2.5 (0–4)	3.0 (2–3)	
Internal Medicine (46)	2.6 (0–4)	3.0 (2–3)	0.735
Pediatrics (25)	2.5 (0–4)	3.0 (2–3)	0.887
Gynecology (72)	2.7 (0–4)	3.0 (2–4)	
Others (102)	2.4 (0–4)	3.0 (2–3)	
**Experience**			
Specialist (130)	2.2 (0–4)	2.0 (1–3)	0.000
Resident (215)	2.6 (0–4)	3.0 (2–3)	0.000
General practitioner (67)	3.0 (0–4)	3.0 (3–4)	
**Work**			
Ministry of health (158)	2.6 (0–4)	3.0 (2–3)	0.514
Academia (128)	2.5 (0–4)	3.0 (2–3)	0.339
Private sector (105)	2.7 (0–4)	3.0 (2–4)	
Military Services (21)	2.8 (1–4)	3.0 (2–3)	

The first p-value shows the ANOVA results for differences between means, while the second p-value shows the difference between medians using Kruskal-Wallis test.

### 3.5. Not having extra marital activity is the main reason chosen by Jordanian physicians who would not take the vaccine themselves

**Fig 2:**
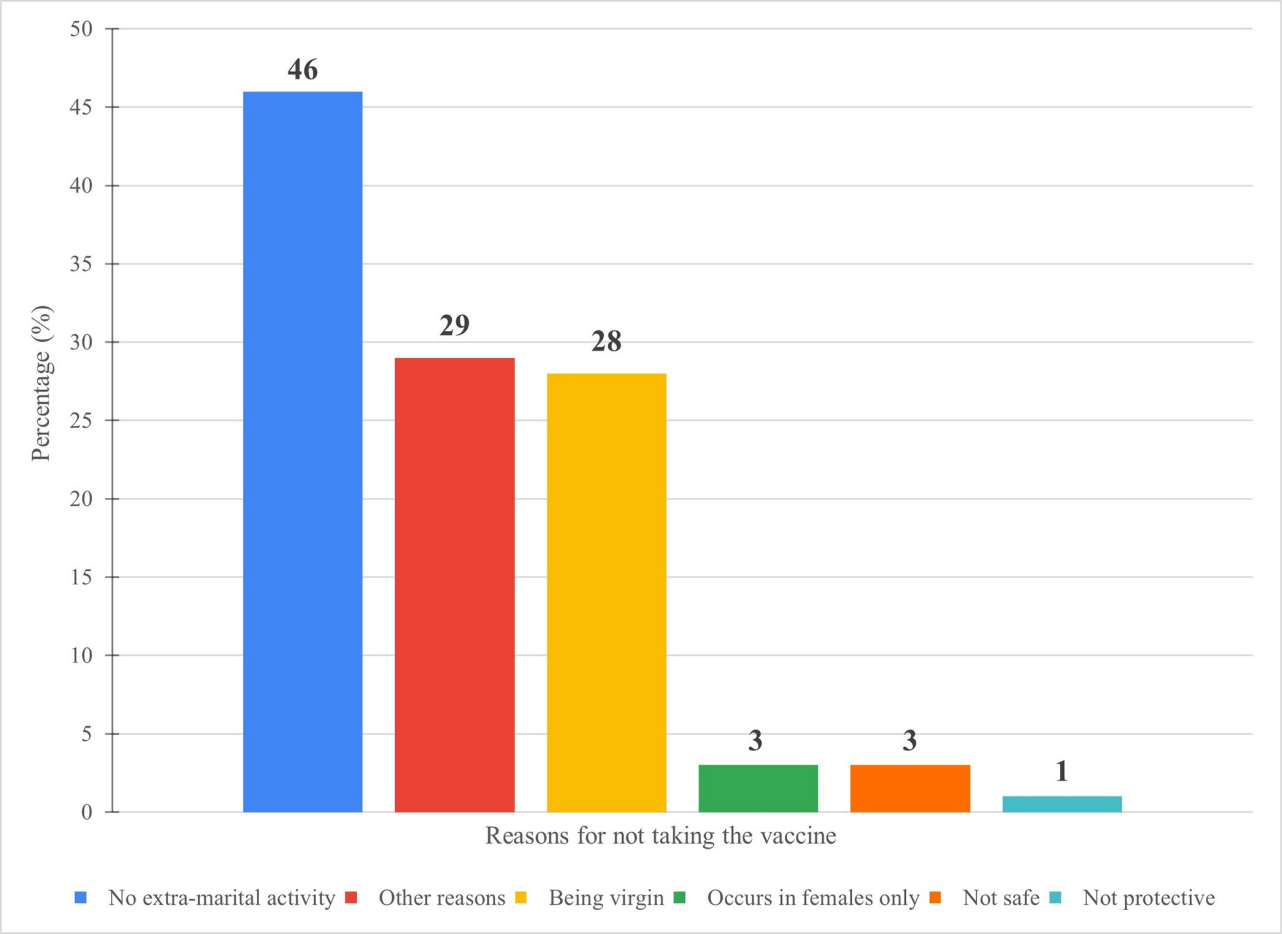
Reasons for not taking the vaccine.

151 of the participants (37%) reported that they won’t take the vaccine if it was available. Not having extra-marital activity was the utmost cause for not taking the HPV vaccine (46%). 28% of the participants won’t take the vaccine because they are virgin, while 3% of the participants won’t take it due to the misconception that HPV occurs in females only. 3% and 1% of the participants believe that the HPV vaccine isn’t safe or protective, respectively. 29% of the participants have other reasons (Fig 2).

### 3.6. Physicians who would not recommend the HPV vaccine to their patients would not do so because they think it is not necessary

**Fig 3:**
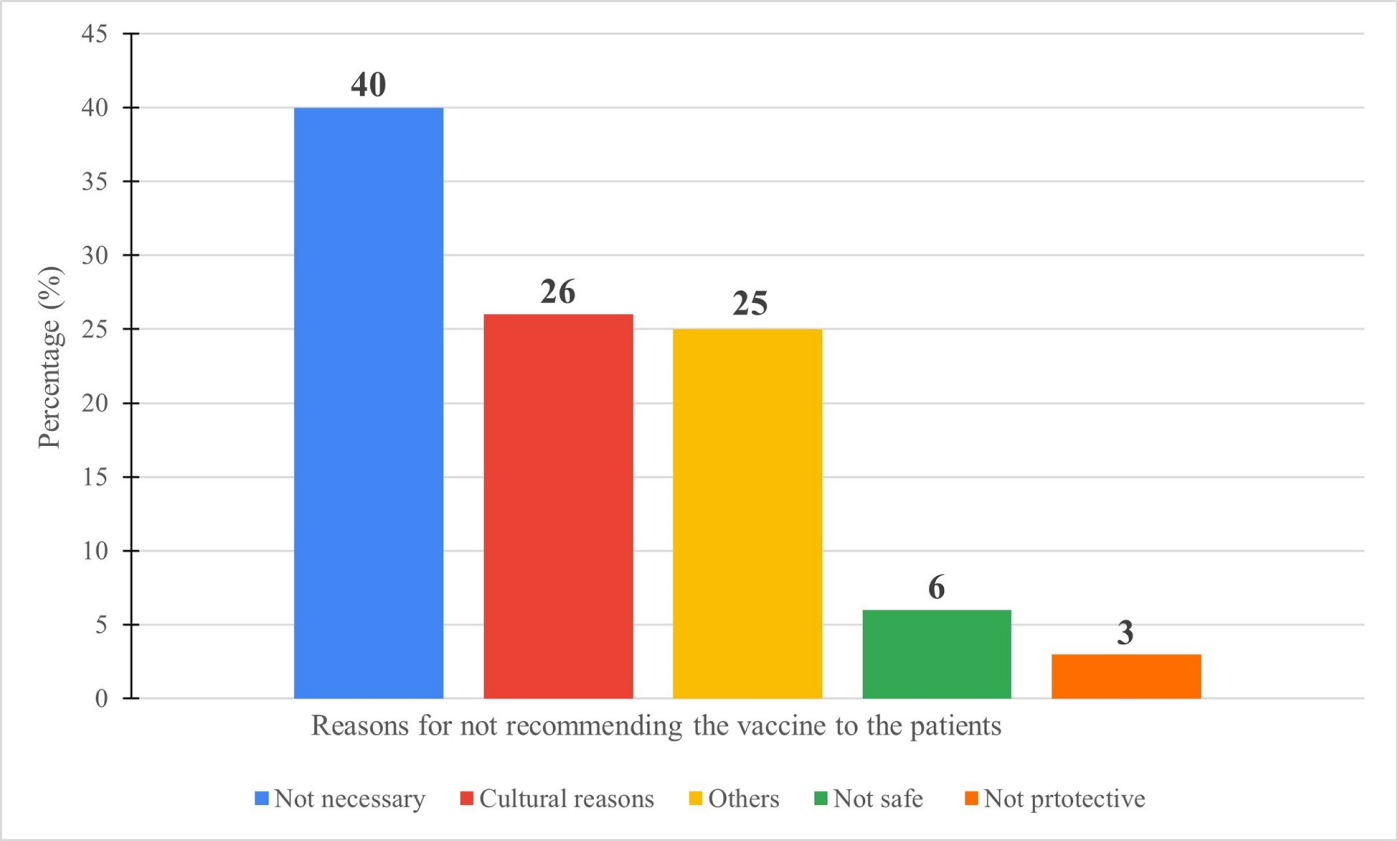
Reasons for not recommending the vaccine to the patients.

Out of 403 participants, 331 (82%) stated that they will recommend the HPV vaccine to their patients if the vaccine was available in Jordan for free, while 72 (18%) won’t. The majority of those who won’t recommend the vaccine (40%) believed that it is not necessary to implement HPV vaccination in Jordan, while 26% won’t recommend it due to cultural reasons. Only 6% and 3% of the physicians won’t recommend the vaccine because it is not safe or not protective, respectively. On the other hand, 25% of the participants justified their “no” answer for other reasons (Fig 3).

### 3.7. Positive attitude of participants towards HPV vaccination correlates with higher knowledge on HPV

**Fig 4:**
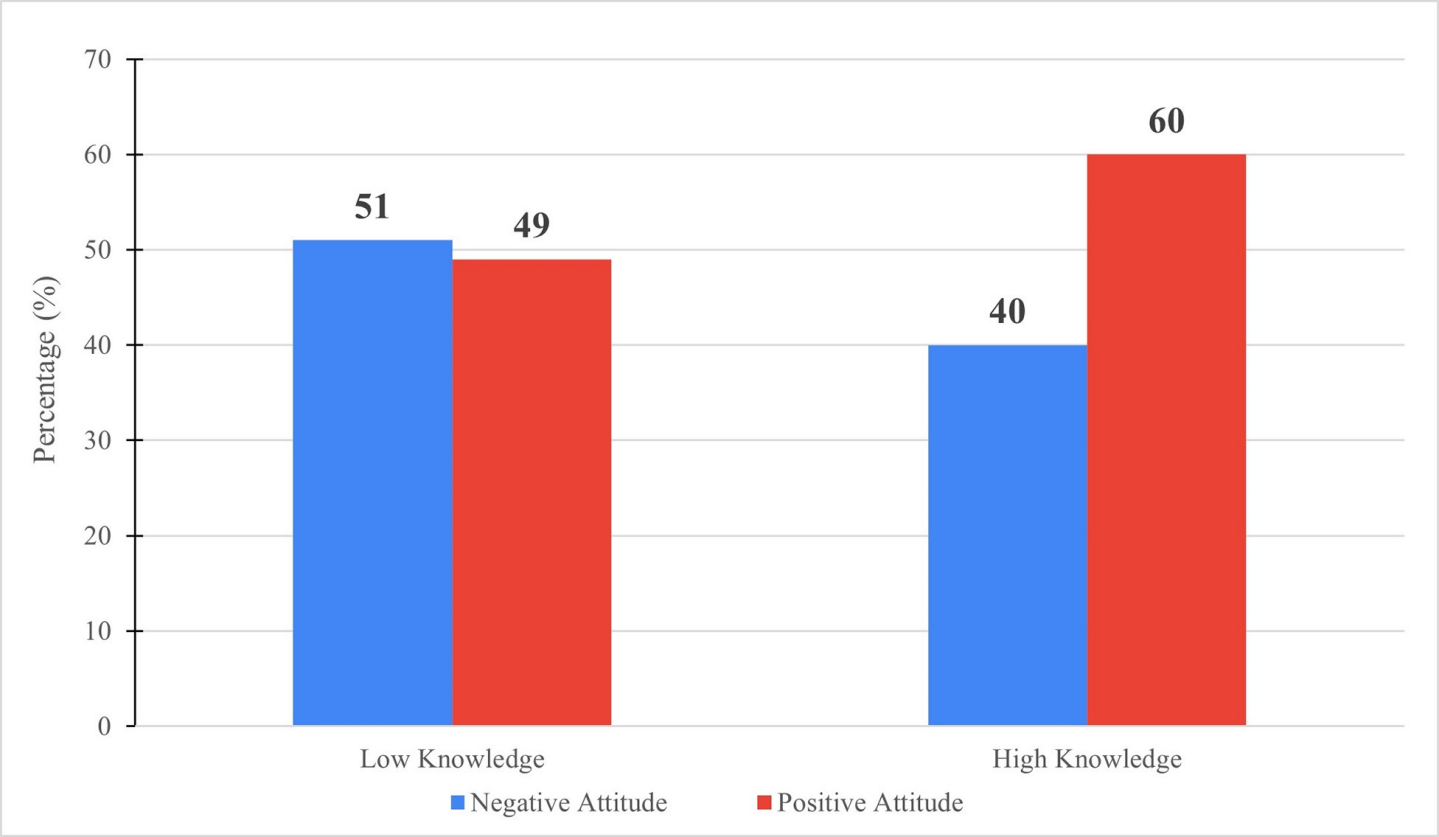
Correlation between total knowledge scores and attitude towards HPV vaccination and screening.

Participants with a K-total score of ≥7.5 out of 15 were classified as “high knowledge” participants and those who got lower than that were classified as “low knowledge” participants. Similarly, based on attitude, physicians with attitude scores ≥3 out of 4 were classified as physicians with “positive attitude” and those who scored <3 out of 4 were classified as physicians with “negative attitude”. A Mann-Whitney test demonstrated a statistically significant difference in terms of attitude towards HPV vaccination and screening among the “high knowledge” but not the “low knowledge” participants (p = 0.039). As shown in Fig 4, 60% of “high knowledge” participants had positive attitude toward HPV screening and vaccination, while those with “low knowledge” had close to equal percentages of negative and positive attitude.

### 3.8. Source of knowledge on HPV vaccines

Among participants who heard about HPV, 93% know about HPV vaccine through medical education and 20% read about HPV vaccine on their own. However, 14% attained their knowledge about HPV vaccine through both, medical education and their own readings.

Social media was the only source of knowledge in 4% of the participants. Friends and family formed the HPV vaccine knowledge source of 10% and 5% of the participants, respectively. Multiple sources of knowledge (three or more) were reported by 1% of the participants, among those 2% reported that they know about HPV vaccine through medical education, their own readings and social media. Good to mention that there is missing data from 77 participants.

## 4. Discussion

This is the first study to shed light on the awareness of Jordanian physicians on the non-sexual routes of HPV transmission. Although penetrative sex is considered the premium cause of HPV infection and genital cancers, non-sexual transmission can add to those cases and to other types of malignancies such as skin, oral and head and neck cancers. Medical equipment that get into direct contact with the genitals and the oral cavity can form major sources of infection, especially if they are not well disinfected to kill the virus. Physicians, dentists and healthcare workers involved in using/disinfecting such equipment must know that HPV cannot be killed by alcohol-based disinfectants and must switch to hypochlorite based ones [8]. In Jordan, as well as many other parts of the world, dermatology and plastic medicine clinics have spread rapidly during the past few years. Very affordable prices of “unwanted hair” removal with laser encouraged females and, to a lower extent, males to perform multiple sessions of hair removal including areas around the genitals. We believe that laser probes have the potential to be a rich source of HPV transmission if not disinfected properly.

Surprisingly, physicians in this study showed extremely low levels of knowledge on non-sexual routes of HPV infection. All in all, out of 5, the average K-nonsexual score of 403 physicians who have previously heard of HPV is ~2.3. Although skin to mucosa and skin to skin contact is a means of sexual transmission of HPV, only 64% and 52% of participants respectively, agreed that these could form an HPV transmission route. Physicians’ disbelief in contaminated water (85%), self-inoculation (77%), medical equipment (66%) and mother to fetus (49%) as potential routes of HPV transmission is worrisome.

In fact, to the best of our knowledge, this is the first published study that reveals the knowledge of physicians on non-sexual routes of HPV transmission as a primary focus. However, a few reports in the literature included questions about non-sexual routes of HPV transmission listed in their questionnaire, but not discussed thoroughly in the text. Results of such reports are very comparable to ours and all express low knowledge of physicians, health professionals and medical students on the non-sexual routes of HPV transmission.

As reported by researchers from Rio de Janeiro state of Brazil, only 27.9% of the participating physicians were able to accurately identify all possible forms of transmission [43]. In Fujian Province, ˃50% of medical staff and students involved in the study did not know that HPV could be transmitted through indirect contact [44]. Similarly, in a study conducted in Greece, only 34% and 20.7% of educators and health professionals knew that HPV can be transmitted through contaminated objects and during natural birth, respectively [45].

Regarding all 3 knowledge categories (K-general, K-nonsexual and K-vaccines), female physicians showed significantly higher scores compared to males. Similarly, females showed better attitude towards HPV vaccination and screening. In fact, this proved to be a general worldwide trend, not only in physicians, but also in students, general population and healthcare workers [46–50]. In Canada, female physicians demonstrated greater knowledge of HPV than their male counterparts [51]. In France, female primary health care (PHC) physicians (78.4%) were found to be more engaged in the performance of Pap smears compared to males (45.7%) [52]. Interestingly, Sallam et. al. (2021) reported that although female medical students had better knowledge on HPV vaccination, male medical students were more aware of HPV association with oral and penile cancers [38]. Hence, this global trend of higher HPV knowledge in females compared to males can be explained by the greatest global association of HPV with cervical cancer as a major cause of female mortality, and the advertised importance of HPV vaccination in preventing it. The role of HPV vaccination in preventing other types of HPV-caused cancers is not publicized [53].

Looking at age, age group ≤ 25 showed significantly higher K-vaccines scores and more positive attitude towards HPV vaccination and screening. In fact, this was reflected by the better attitude of GPs, as most of our GPs in this study are fresh graduates who belong the youngest age group. This is non-consistent with previous literature from Saudi Arabia where younger (20–30 years old) participants scored the lowest regarding HPV knowledge [54]. On the contrary, another Saudi study reported that physicians’ age was not associated with knowledge of cervical cancer or HPV [55]. The younger age-higher knowledge-more positive attitude finding of this study could reflect updated universities’ curricula, fresher and more generalized knowledge of new graduates and/or more engagement with globalization and international openness.

Academic physicians showed significantly greater knowledge than their MOH, private practice and MRS counterparts. Despite the fact that physicians engaged with academic work might spend less time in the clinic and might have less direct contact with patients, they proved to know better about HPV in general and about HPV vaccines compared to their clinical fellows. Accordingly, this better knowledge reflected on the attitudes of academic physicians towards HPV vaccination and screening. Interestingly, in India, community physicians demonstrated better basic HPV knowledge compared to academic physicians, whereas academic physicians showed better vaccine knowledge and more positive vaccination attitudes [56]. The better HPV knowledge and more positive attitude towards HPV vaccination and screening detected in Jordanian academic physicians in this study could be explained by the continuous and updated reading demands of the academic work. In addition, academic doctors are more exposed to scientific communication and outreaches through national and international scientific conferences, seminars and journal clubs. This emphasizes on the importance of continuous training and education and on the self-driven reading on updates in medical fields of special and public importance.

Based on specialty, no statistically significant difference was found between physicians with different specialties in terms of knowledge and attitudes towards HPV vaccination and screening. Noteworthy, although not significant, gynecologists showed higher K-vaccine scores compared to surgeons, pediatricians, internists and other specialty physicians (P = 0.059). This lack of discrepancies between specialties regarding HPV knowledge and vaccination was reported by a recent study in Israel [57, 58]. Farah et. al., (2022) reported no difference in HPV-related knowledge among gynecologists, pediatricians, and internal medicine doctors. On the other hand, a study from Solvenia observed a significantly higher HPV knowledge and more positive attitude toward HPV vaccination among pediatricians and school medicine specialists [59]. Similarly, in Saudi Arabia, better knowledge was scored by obstetrics-gynecologists [54] and family medicine physicians [55] compared to their counterparts.

In terms of experience, general practitioners, residents and specialists showed no difference in all measures of HPV knowledge as well as attitude towards vaccination and screening. Similar results were reported by scholarly articles in the literature. In Israel, no difference was found between residents and specialists regarding HPV related knowledge [57]. Similarly, a study performed in Louisiana, United States couldn’t link between knowledge and years of experience [58]. On the other hand, in Saudi Arabia, higher practice level showed a significant impact on knowledge strength as reflected by higher scores [54, 55].

Limitations of this study included the use of a convenience sampling approach, with the possibility of selection bias. Thus, the generalizability of the results should be approached with caution. Careless or multiple responses can’t be ruled out. As noted with many older physicians, and physicians with specialties not much related to HPV infections (for example: ophthalmologists, orthopedics and others) affected their decision on participating in the survey based on their lack of previous knowledge of the survey subject. Thus, the genuine level of knowledge among physicians can be lower than the level reported in this study. In addition, this survey study was conducted just after the COVID-19 crisis started to ease down, at times preceded by a tremendous amount of COVID-19 related survey based research in the country [60]. Physicians were overwhelmed with the amount of survey invitations they had received and were discouraged to participate in any study. Another issue reported by researchers conducting the in-person interviews is the way physicians treated questionnaire questions as if they were exam questions. Physicians sought to give the right answers rather than being honest with what they know about the study subject.

## 5. Conclusions

The noteworthy findings of this study that should be highlighted include: 1- The extremely low level of knowledge on non-sexual routes of HPV infection among Jordanian physicians. 2- The significantly higher K-general, K-nonsexual and K-vaccines scores and better attitude towards HPV vaccination and screening reflected by female physicians compared to their male counterparts. 3- The stronger K-vaccines score of the youngest physicians (age group ≤ 25). 4- The significantly greater HPV knowledge and more positive attitude of academic physicians compared to their MOH, private practice and MRS fellows. 5-The lack of specialty and practice level influence on the knowledge and attitudes of participants.

Increasing the level of awareness of physicians and healthcare workers on the non-sexual routes of HPV transmission and their association with cervical and other cancers through university curricula and other reliable sources is recommended. This must include proper ways of disinfecting medical equipment to eliminate contaminating HPV. Educating the community through socio-educational campaigns on these nonsexual routes can help break the religious/cultural barrier towards HPV infection. This can help in the acceptance and implementation of HPV vaccination and screening; in hopes to help in the prevention of HPV associated cancers.

## Supporting information

S1 Table Supplementary tables participants. * Out of 412; ** out of 403.
(DOCX)

S2 Table Participants answers to knowledge assessment questionnaire about HPV. ** out of 403.
(DOCX)

S3 Table Participants knowledge about transmission of HPV. ** out of 403.
(DOCX)

S4 Table Assessment of participants attitude toward HPV screening and testing. ** out of 403.
(DOCX)

S5 Table Participants knowledge about HPV vaccine and their opinions about vaccines in Jordan. ** out of 403; *** out of 332.
(DOCX)

S6 Table Knowledge score calculation. (DOCX)

S1 File (XLSX)

## References

[1] Dunne E.F. and Park I.U., HPV and HPV-associated diseases. Infect Dis Clin North Am, 2013. 27(4): p. 765–78. doi: 10.1016/j.idc.2013.09.001 24275269

[2] de Martel C., et al., Worldwide burden of cancer attributable to HPV by site, country and HPV type. Int J Cancer, 2017. 141(4): p. 664–670. doi: 10.1002/ijc.30716 28369882 PMC5520228

[3] Crosbie E.J., et al., Human papillomavirus and cervical cancer. Lancet, 2013. 382(9895): p. 889–99. doi: 10.1016/S0140-6736(13)60022-7 23618600

[4] Graham S.V., The human papillomavirus replication cycle, and its links to cancer progression: a comprehensive review. Clin Sci (Lond), 2017. 131(17): p. 2201–2221. doi: 10.1042/CS20160786 28798073

[5] Rosales R. and Rosales C., Immune therapy for human papillomaviruses-related cancers. World J Clin Oncol, 2014. 5(5): p. 1002–19. doi: 10.5306/wjco.v5.i5.1002 25493236 PMC4259927

[6] Alizon S., Murall C.L., and Bravo I.G., Why Human Papillomavirus Acute Infections Matter. Viruses, 2017. 9(10). doi: 10.3390/v9100293 28994707 PMC5691644

[7] Ferenczy A. and Franco E., Persistent human papillomavirus infection and cervical neoplasia. Lancet Oncol, 2002. 3(1): p. 11–6. doi: 10.1016/s1470-2045(01)00617-9 11905599

[8] Petca A., et al., Non-sexual HPV transmission and role of vaccination for a better future (Review). Exp Ther Med, 2020. 20(6): p. 186. doi: 10.3892/etm.2020.9316 33101476 PMC7579832

[9] Liu Z., Rashid T., and Nyitray A.G., Penises not required: a systematic review of the potential for human papillomavirus horizontal transmission that is non-sexual or does not include penile penetration. Sex Health, 2016. 13(1): p. 10–21. doi: 10.1071/SH15089 26433493

[10] Tay S.K., Ho T.H., and Lim-Tan S.K., Is genital human papillomavirus infection always sexually transmitted? Aust N Z J Obstet Gynaecol, 1990. 30(3): p. 240–2. doi: 10.1111/j.1479-828x.1990.tb03223.x 2256864

[11] Mammas I.N., et al., Paediatric virology and human papillomaviruses: An update. Exp Ther Med, 2019. 17(6): p. 4337–4343. doi: 10.3892/etm.2019.7516 31186676 PMC6507507

[12] Hong Y., et al., Survey of human papillomavirus types and their vertical transmission in pregnant women. BMC Infectious Diseases, 2013. 13(1): p. 109. 23446269 10.1186/1471-2334-13-109 PMC3598550

[13] Ryndock E.J. and Meyers C., A risk for non-sexual transmission of human papillomavirus? Expert Rev Anti Infect Ther, 2014. 12(10): p. 1165–70. doi: 10.1586/14787210.2014.959497 25199987

[14] Fratini M., Di Bonito P., and La Rosa G., Oncogenic Papillomavirus and Polyomavirus in Water Environments: Is There a Potential for Waterborne Transmission? Food Environ Virol, 2014. 6(1): p. 1–12. doi: 10.1007/s12560-013-9134-0 24293168

[15] Iaconelli M., et al., First Detection of Human Papillomaviruses and Human Polyomaviruses in River Waters in Italy. Food Environ Virol, 2015. 7(4): p. 309–15. doi: 10.1007/s12560-015-9203-7 26049729

[16] Rose J. and Jiménez-Cisneros B., Global Water Pathogen Project. East Lansing, MI: Michigan State Univ, 2019.

[17] Gallay C., et al., Human papillomavirus (HPV) contamination of gynaecological equipment. Sexually transmitted infections, 2016. 92(1): p. 19–23. doi: 10.1136/sextrans-2014-051977 26071392

[18] Galati L., et al., HPV and head and neck cancers: Towards early diagnosis and prevention. Tumour Virus Research, 2022: p. 200245. doi: 10.1016/j.tvr.2022.200245 35973657 PMC9420391

[19] Bruni L., et al., Cervical human papillomavirus prevalence in 5 continents: meta-analysis of 1 million women with normal cytological findings. Journal of Infectious Diseases, 2010. 202(12): p. 1789–1799. doi: 10.1086/657321 21067372

[20] Al‐Awadhi R., Chehadeh W., and Kapila K., Prevalence of human papillomavirus among women with normal cervical cytology in Kuwait. Journal of medical virology, 2011. 83(3): p. 453–460. doi: 10.1002/jmv.21981 21264866

[21] Elmi A.A., et al., Human papillomavirus (HPV) infection: molecular epidemiology, genotyping, seroprevalence and associated risk factors among Arab women in Qatar. PloS one, 2017. 12(1): p. e0169197. doi: 10.1371/journal.pone.0169197 28046025 PMC5207789

[22] AlObaid A., et al., Human papillomavirus prevalence and type distribution among women attending routine gynecological examinations in Saudi Arabia. BMC infectious diseases, 2014. 14(1): p. 1–8. doi: 10.1186/s12879-014-0643-8 25496614 PMC4272558

[23] Moosa K., et al., An epidemiological study assessing the prevalence of human papillomavirus types in women in the Kingdom of Bahrain. BMC cancer, 2014. 14: p. 1–8. 25466757 10.1186/1471-2407-14-905 PMC4265506

[24] Lubbad A.M. and Al-Hindi A., Bacterial, viral and fungal genital tract infections in Palestinian pregnant women in Gaza, Palestine. West African Journal of Medicine, 2007. 26(2): p. 138–142. 17939317

[25] Mahafzah A.M., et al., Prevalence of sexually transmitted infections among sexually active Jordanian females. Sex Transm Dis, 2008. 35(6): p. 607–10. doi: 10.1097/OLQ.0b013e3181676bbd 18434942

[26] Maraqa B., et al., Prevalence of abnormal pap smears: a descriptive study from a Cancer Center in a low-prevalence community. Asian Pacific journal of cancer prevention: APJCP, 2017. 18(11): p. 3117. doi: 10.22034/APJCP.2017.18.11.3117 29172288 PMC5773800

[27] Khasawneh A.I., et al., Prevalence and Genotype Distribution of Human Papillomavirus Among a Subpopulation of Jordanian Women. International Journal of Women’s Health and Reproduction Sciences, 2021. 9(1): p. 17–23.

[28] Sharkas G., et al., Trends in the incidence of cervical cancer in Jordan, 2000–2013. Journal of Oncology, 2017. 2017. doi: 10.1155/2017/6827384 28932241 PMC5592005

[29] Al-Sayaideh A., et al., Cancer Incidence In Jordan—2012. 2016.

[30] Jordan T.H.I.C. Human Papillomavirus and Related Cancers, Fact Sheet 2021. 2021; Available from: https://hpvcentre.net/statistics/reports/JOR_FS.pdf.

[31] Abu-Lubad M.A., et al., Human papillomavirus as an independent risk factor of invasive cervical and endometrial carcinomas in Jordan. Journal of Infection and Public health, 2020. 13(4): p. 613–618. doi: 10.1016/j.jiph.2019.08.017 31519382

[32] Obeidat B., et al., Prevalence and distribution of high-risk human papillomavirus genotypes in cervical carcinoma, low-grade, and high-grade squamous intraepithelial lesions in Jordanian women. European journal of gynaecological oncology, 2013. 34(3): p. 257–260. 23967558

[33] Qatouseh L.A., et al., Detection of high-risk human papillomavirus genotypes 16 and 18 in head and neck squamous cell carcinomas in Jordan. Asian Pacific journal of cancer prevention: APJCP, 2017. 18(5): p. 1337. doi: 10.22034/APJCP.2017.18.5.1337 28612284 PMC5555544

[34] Khasawneh A.I., et al., Prevalence of human papillomavirus associated with head and neck squamous cell carcinoma in Jordanian patients. The Open Microbiology Journal, 2020. 14(1).

[35] Asali F., et al., Jordanian women’s attitudes towards cervical cancer screening: has the pattern changed? Journal of Obstetrics and Gynaecology, 2020. 40(4): p. 564–568. doi: 10.1080/01443615.2019.1635097 31478428

[36] Amarin Z., Badria L., and Obeidat B., Attitudes and beliefs about cervical smear testing in ever-married Jordanian women. EMHJ-Eastern Mediterranean Health Journal, 14 (2), 389–397, 2008, 2008. 18561732

[37] Obeidat B., Amarin Z., and Alzaghal L., Awareness, practice and attitude to cervical Papanicolaou smear among female health care workers in Jordan. European journal of cancer care, 2012. 21(3): p. 372–376. doi: 10.1111/j.1365-2354.2011.01297.x 22050559

[38] Sallam M., et al., Attitude towards HPV Vaccination and the Intention to Get Vaccinated among Female University Students in Health Schools in Jordan. Vaccines (Basel), 2021. 9(12). doi: 10.3390/vaccines9121432 34960177 PMC8707789

[39] Alsous M.M., et al., Knowledge about cervical cancer and awareness about human papillomavirus vaccination among medical students in Jordan. PeerJ, 2021. 9: p. e11611. doi: 10.7717/peerj.11611 34178471 PMC8214844

[40] Lataifeh I., et al., A survey of Jordanian obstetricians and gynecologists’ knowledge and attitudes toward human papillomavirus infection and vaccination. European journal of gynaecological oncology, 2014. 35(4): p. 429–432. 25118486

[41] Sallam M., et al., Lack of knowledge regarding HPV and its relation to oropharyngeal cancer among medical students. Cancer Reports, 2022. 5(7): p. e1517. doi: 10.1002/cnr2.1517 34291614 PMC9327668

[42] Sallam M., et al., Dental students’ awareness and attitudes toward HPV-related oral cancer: a cross sectional study at the University of Jordan. BMC oral health, 2019. 19: p. 1–11. doi: 10.1186/s12903-019-0864-8 31370845 PMC6670240

[43] Mello V., et al., Knowledge about human papillomavirus transmission and prevention among physicians in Rio de Janeiro state, Brazil. Rev Assoc Med Bras (1992), 2023. 69(4): p. e20220291. 10.1590/1806-9282.20220291 PMC10176655 37098929

[44] Yu C., et al., Evaluation of Knowledge and Attitude Toward HPV and Vaccination Among Medical Staff, Medical Students, and Community Members in Fujian Province. Risk Manag Healthc Policy, 2020. 13: p. 989–997. doi: 10.2147/RMHP.S243048 32801973 PMC7413698

[45] Xenaki D., et al., Knowledge, behaviours and attitudes for human papillomavirus (HPV) prevention among educators and health professionals in Greece. Eur Rev Med Pharmacol Sci, 2020. 24(14): p. 7745–7752. doi: 10.26355/eurrev_202007_22277 32744701

[46] Gamaoun R., Knowledge, awareness and acceptability of anti-HPV vaccine in the Arab states of the Middle East and North Africa Region: a systematic. EMHJ, 2018. 24(6–2018). 10.26719/2018.24.6.538 30079949

[47] Chen G., et al., Gender differences in knowledge and attitude towards HPV and HPV vaccine among college students in Wenzhou, China. Vaccines, 2021. 10(1): p. 10. doi: 10.3390/vaccines10010010 35062671 PMC8779512

[48] Indracanti M., Berhane N., and Minyamer T., Factors Associated with Pre-and Post-Educational Intervention Knowledge Levels of HPV and Cervical Cancer Among the Male and Female University Students, Northwest Ethiopia. Cancer Management and Research, 2021: p. 7149–7163. doi: 10.2147/CMAR.S326544 34548819 PMC8449546

[49] Preston S.M. and Darrow W.W., Are men being left behind (or catching up)? Differences in HPV awareness, knowledge, and attitudes between diverse college men and women. American Journal of Men’s Health, 2019. 13(6): p. 1557988319883776. 10.1177/1557988319883776 PMC6887835 31787066

[50] Neugut A.I., et al., Physician characteristics and decisions regarding cancer screening: a systematic review. Population Health Management, 2019. 22(1): p. 48–62. doi: 10.1089/pop.2017.0206 29889616

[51] Steben M., et al., A National Survey of Canadian physicians on HPV: knowledge, barriers, and preventive practices. Journal of Obstetrics and Gynaecology Canada, 2019. 41(5): p. 599–607. e3. doi: 10.1016/j.jogc.2018.09.016 30595515

[52] Rochoy M., et al., Factors associated with the achievement of cervical smears by general practitioners. BMC research notes, 2017. 10(1): p. 1–5. 29221494 10.1186/s13104-017-2999-5 PMC5723048

[53] Shapiro G.K., HPV Vaccination: an underused strategy for the prevention of cancer. Current Oncology, 2022. 29(5): p. 3780–3792. doi: 10.3390/curroncol29050303 35621693 PMC9140027

[54] Anfinan N.M., Physician’s knowledge and opinions on human papillomavirus vaccination: a cross-sectional study, Saudi Arabia. BMC health services research, 2019. 19(1): p. 1–12. 31830983 10.1186/s12913-019-4756-z PMC6909584

[55] Almazrou S., Saddik B., and Jradi H., Knowledge, attitudes, and practices of Saudi physicians regarding cervical cancer and the human papilloma virus vaccine. Journal of infection and public health, 2020. 13(4): p. 584–590. doi: 10.1016/j.jiph.2019.09.002 31570271

[56] Canon C., et al., Knowledge and attitudes towards human papillomavirus (HPV) among academic and community physicians in Mangalore, India. Journal of Cancer Education, 2017. 32: p. 382–391. 26880357 10.1007/s13187-016-0999-0

[57] Khamisy-Farah R., et al., Knowledge of human papillomavirus (HPV), attitudes and practices towards anti-HPV vaccination among Israeli pediatricians, gynecologists, and internal medicine doctors: development and validation of an ad hoc questionnaire. Vaccines, 2019. 7(4): p. 157. doi: 10.3390/vaccines7040157 31640127 PMC6963669

[58] Mehta V., et al., Knowledge of HPV-related oropharyngeal cancer and use of human papillomavirus vaccines by pediatricians in Louisiana. J La State Med Soc, 2017. 169(2): p. 37–42. 28414659

[59] Troha M., et al., Human papillomavirus (HPV) infection and vaccination: Knowledge and attitudes among healthcare professionals and the general public in Slovenia. Acta Dermatovenerol Alp Pannonica Adriat, 2018. 27(2): p. 59–64. 29945260

[60] Qaqish A., Al-Omari M., and Dajani R., Obstacles in the road of biomedical research on COVID-19 in Jordan: Poor funding and beyond. Journal of Global Health, 2022. 12. doi: 10.7189/jogh.12.03052 35841621 PMC9288256

