# Supplementary material for: Awareness and knowledge of physicians and residents on the non-sexual routes of human papilloma virus (HPV) infection and their perspectives on anti-HPV vaccination in Jordan
Source: PLoS One. 2023 Oct 11;18(10):e0291643. doi: 10.1371/journal.pone.0291643 (PMC10566688; doi:10.1371/journal.pone.0291643)
Supplement: S1 Table — * Out of 412; ** out of 403. (DOCX) [file pone.0291643.s001.docx]

S1: Supplementary tables Participants

| **Factor** | **Number** | **%** |
| --- | --- | --- |
| **Previous training regarding STI*** |  |  |
| Yes | 80 | 19.4 |
| No | 332 | 80.5 |
| **Participation in research regarding STI*** |  |  |
| Yes | 21 | 5.1 |
| No | 391 | 94.9 |
| **Heard about HPV*** |  |  |
| Yes | 403 | 97.8 |
| No | 9 | 2.2 |
| **Encounter HPV patients**** |  |  |
| Never | 217 | 53.8 |
| Rarely | 119 | 29.5 |
| Occasionally | 47 | 11.7 |
| Often | 20 | 5 |
| **HPV is common in Jordan**** |  |  |
| Yes | 93 | 23.1 |
| No | 133 | 33 |
| I don’t know | 177 | 43.9 |

* Out of 412

** out of 403
