# Supplementary material for: Awareness and knowledge of physicians and residents on the non-sexual routes of human papilloma virus (HPV) infection and their perspectives on anti-HPV vaccination in Jordan
Source: PLoS One. 2023 Oct 11;18(10):e0291643. doi: 10.1371/journal.pone.0291643 (PMC10566688; doi:10.1371/journal.pone.0291643)
Supplement: S2 Table — ** out of 403. (DOCX) [file pone.0291643.s002.docx]

S2:Participants answers to knowledge assessment questionnaire about HPV

| **Factor **** | **Number** | **%** |
| --- | --- | --- |
| **Gender affected by HPV** |  |  |
| Both males and females | 389 | 96.5 |
| Females only | 8 | 2 |
| Males only | 0 | 0 |
| I don’t know | 6 | 1.5 |
| **HPV infection can be Asymptomatic** |  |  |
| Yes | 359 | 89.1 |
| No | 15 | 3.7 |
| I don’t know | 29 | 7.2 |
| **Most Adverse Complication of HPV** |  |  |
| In Females |  |  |
| Cancer | 301 | 74.7 |
| Genital warts | 84 | 20.8 |
| Skin warts | 12 | 3 |
| Others | 6 | 1.5 |
| In Males |  |  |
| Cancer | 106 | 26.3 |
| Genital warts | 238 | 59.1 |
| Skin warts | 44 | 10.9 |
| Others | 15 | 3.7 |
| **HPV can be treated** |  |  |
| Yes | 326 | 80.9 |
| No | 57 | 14.1 |
| I don’t know | 20 | 5 |

** out of 403
