# Supplementary material for: Awareness and knowledge of physicians and residents on the non-sexual routes of human papilloma virus (HPV) infection and their perspectives on anti-HPV vaccination in Jordan
Source: PLoS One. 2023 Oct 11;18(10):e0291643. doi: 10.1371/journal.pone.0291643 (PMC10566688; doi:10.1371/journal.pone.0291643)
Supplement: S3 Table — ** out of 403. (DOCX) [file pone.0291643.s003.docx]

S3: Participants knowledge about Transmission of HPV

| **Route of Transmission **** | **Number** | **%** |
| --- | --- | --- |
| **Sexual intercourse** |  |  |
| Yes | 391 | 97 |
| No | 12 | 3 |
| **Skin to skin contact** |  |  |
| Yes | 209 | 51.9 |
| No | 194 | 48.1 |
| **Skin to mucosa contact** |  |  |
| Yes | 257 | 63.8 |
| No | 146 | 36.2 |
| **Mother to fetus** |  |  |
| Yes | 207 | 51.4 |
| No | 196 | 48.6 |
| **Contaminated medical equipment** |  |  |
| Yes | 139 | 34.5 |
| No | 264 | 65.5 |
| **Self-inoculation** |  |  |
| Yes | 91 | 22.6 |
| No | 312 | 77.4 |
| **Contaminated water** |  |  |
| Yes | 61 | 15.1 |
| No | 342 | 84.9 |

** out of 403
