# Supplementary material for: Awareness and knowledge of physicians and residents on the non-sexual routes of human papilloma virus (HPV) infection and their perspectives on anti-HPV vaccination in Jordan
Source: PLoS One. 2023 Oct 11;18(10):e0291643. doi: 10.1371/journal.pone.0291643 (PMC10566688; doi:10.1371/journal.pone.0291643)
Supplement: S4 Table — ** out of 403. (DOCX) [file pone.0291643.s004.docx]

S4: Assessment of participants attitude toward HPV screening and testing

| **Factor **** | **Number** | **%** |
| --- | --- | --- |
| **Participant recommend regular testing of HPV, especially among young females** |  |  |
| Yes | 310 | 76.9 |
| No | 22 | 23.1 |
| **Participant recommend regular testing of HPV, among high school students** |  |  |
| Yes | 147 | 36.5 |
| No | 256 | 63.5 |
| **Participant believes that the community lacks enough knowledge about HPV in Jordan** |  |  |
| Yes | 399 | 99 |
| No | 4 | 1 |
| **Participant believes that the Physicians lack adequate knowledge about HPV in Jordan** |  |  |
| Yes | 367 | 91.1 |
| No | 36 | 8.9 |

** out of 403
