# Supplementary material for: Awareness and knowledge of physicians and residents on the non-sexual routes of human papilloma virus (HPV) infection and their perspectives on anti-HPV vaccination in Jordan
Source: PLoS One. 2023 Oct 11;18(10):e0291643. doi: 10.1371/journal.pone.0291643 (PMC10566688; doi:10.1371/journal.pone.0291643)
Supplement: S5 Table — ** out of 403; *** out of 332. (DOCX) [file pone.0291643.s005.docx]

S5: Participants knowledge about HPV vaccine and their opinions about vaccines in Jordan

| **Factor** | **Number** | **%** |
| --- | --- | --- |
| **There is a vaccine available for HPV**** |  |  |
| Yes | 332 | 82.3 |
| No | 38 | 9.4 |
| I don’t know | 33 | 8.2 |
| **Vaccine be protective if the patient is already infected with HPV***** |  |  |
| Yes | 104 | 31.3 |
| No | 154 | 46.4 |
| I don’t know | 74 | 22.3 |
| **Vaccine protection percentage against cervical cancer***** |  |  |
| 25% | 21 | 6.3 |
| 50% | 18 | 5.4 |
| 60% | 62 | 18.6 |
| 90% | 140 | 42.2 |
| I don’t know | 91 | 27.4 |
| **Most appropriate age for HPV vaccination***** |  |  |
| Childhood | 42 | 12.7 |
| Puberty | 112 | 33.7 |
| Prior to marriage/sexual activity | 143 | 43.1 |
| At any age | 18 | 5.4 |
| I don’t know | 17 | 5.1 |
| **HPV vaccine is available in Jordan***** |  |  |
| Yes | 129 | 38.6 |
| No | 38 | 11.4 |
| I don’t know | 165 | 49.7 |
| **HPV vaccination should be obligatory in Jordan***** |  |  |
| Yes | 240 | 59.6 |
| No | 163 | 40.4 |

** out of 403

*** out of 332
