# Supplementary material for: Awareness and knowledge of physicians and residents on the non-sexual routes of human papilloma virus (HPV) infection and their perspectives on anti-HPV vaccination in Jordan
Source: PLoS One. 2023 Oct 11;18(10):e0291643. doi: 10.1371/journal.pone.0291643 (PMC10566688; doi:10.1371/journal.pone.0291643)
Supplement: S6 Table — (DOCX) [file pone.0291643.s006.docx]

S6: Knowledge score calculation

| **Factor** | **Score point** |
| --- | --- |
| **General HPV knowledge** |  |
| Ability of HPV to infect |  |
| Both females and males | 1 |
| Females only | 0 |
| Males only | 0 |
| I don’t know | 0 |
| Asymptomatic HPV infections are possible |  |
| Yes | 1 |
| No | 0 |
| I don’t know | 0 |
| HPV can transmit through sexual routes |  |
| Yes | 1 |
| No | 0 |
| Most serious consequence of HPV infection in females and males |  |
| Cancer, Cancer | 2 |
| Cancer, Genital warts | 1 |
| Other choices * | 0 |
| HPV infections can be treatable |  |
| Yes | 1 |
| No | 0 |
| I don’t know | 0 |
| **Non-sexual HPV knowledge** |  |
| Transmission of HPV through skin to skin |  |
| Yes | 1 |
| No | 0 |
| Transmission of HPV through skin to mucosa |  |
| Yes | 1 |
| No | 0 |
| Mother to fetus transmission of HPV |  |
| Yes | 1 |
| No | 0 |
| HPV transmit through contaminated medical equipments |  |
| Yes | 1 |
| No | 0 |
| HPV transmission through water |  |
| Yes | 0.5 |
| No | 0 |
| Spread of HPV by self-inoculation |  |
| Yes | 0.5 |
| No | 0 |
| **HPV vaccine knowledge** |  |
| There is a vaccine available for HPV |  |
| Yes | 1 |
| No | 0 |
| I don’t know | 0 |
| HPV vaccines are protective against cervical cancer even in those already infected with HPV |  |
| Yes | 1 |
| No | 0 |
| I don’t know | 0 |
| HPV vaccines protection against cervical cancer |  |
| 25% | 0 |
| 50% | 0 |
| 60% | 0 |
| 90% | 1 |
| I don’t know | 0 |
| Most appropriate age for HPV vaccination |  |
| During childhood years | 0 |
| During puberty | 1 |
| Prior to marriage/sexual activity | 1 |
| At any age | 0 |
| I don’t know | 0 |
